# CircRNA CDR1as/miR-641/HOXA9 pathway regulated stemness contributes to cisplatin resistance in non-small cell lung cancer (NSCLC)

**DOI:** 10.1186/s12935-020-01390-w

**Published:** 2020-07-06

**Authors:** Yongsheng Zhao, Renyan Zheng, Jian Chen, Dong Ning

**Affiliations:** 1 Department of Thoracic Surgery, Affiliated Hospital of North Sichuan Medical College, Maoyuan South Road, No. 1, Nanchong, 637000 Sichuan China; 2 Department of Anorectal Medicine, Affiliated Hospital of North Sichuan Medical College, Maoyuan South Road, No. 1, Nanchong, 637000 Sichuan China

**Keywords:** Cisplatin, Non-small cell lung cancer, Cancer stem cells, Chemoresistance

## Abstract

**Background:**

Cisplatin (DDP) is the first-line chemotherapeutic drug for non-small cell lung cancer (NSCLC), and long-term DDP stimulation increased resistance of NSCLC cells to this drug by enriching cancer stem cells (CSCs), which contributed to recurrence and worse prognosis of NSCLC, but the molecular mechanisms are still not fully delineated.

**Methods:**

Real-Time qPCR and Western Blot analysis were conducted to examine gene expressions at mRNA and protein levels, respectively. Dual-luciferase reporter gene system was used to validate the targeting sites among circRNA CDR1as, miR-641 and HOXA9 mRNA. Cell growth was evaluated by CCK-8 assay, trypan blue staining assay and colony formation assay. The Annexin V-FITC/PI double staining method was employed to measure cell apoptosis ratio. Spheroid formation and flow cytometer assay was used to evaluate cell stemness. Xenograft mice models were established to measure tumorgenicity in vivo, and Ki67 expressions in mice tumor tissues were examined by immunohistochemistry (IHC).

**Results:**

Here we identified a novel circRNA CDR1as/miR-641/Homeobox protein Hox-A9 (HOXA9) pathway regulated stemness and DDP chemoresistance in NSCLC. Mechanistically, circRNA CDR1as and HOXA9 were high-expressed, while miR-641 was low-expressed in DDP-resistant NSCLC cells, instead of their corresponding parental DDP-sensitive NSCLC cells. Additionally, we validated that circRNA CDR1as positively regulated HOXA9 in NSCLC cells by serving as an RNA sponge for miR-641, and knock-down of circRNA CDR1as increased the sensitivity of DDP-resistant NSCLC cells, which were reversed by downregulating miR-641 and upregulating HOXA9. Consistently, overexpression of circRNA CDR1as increased drug resistance of DDP-sensitive NSCLC cells by regulating miR-641/HOXA9 axis. In addition, the expression levels of stemness signatures (SOX2, OCT4 and Nanog) were higher in DDP-resistant NSCLC cells, which also tended to form spheres and enrich CD44^+^CD166^+^ population compared to their parental DDP-sensitive NSCLC cells, suggesting that CSCs were enriched in DDP-resistant NSCLC cells. Notably, knock-down of circRNA CDR1as inhibited stemness of DDP-resistant NSCLC cells by inhibiting HOXA9 through upregulating miR-641.

**Conclusions:**

Taken together, this study identified that circRNA CDR1as regulated stemness and DDP chemoresistance in NSCLC cells by targeting miR-641/HOXA9 axis.

## Background

Currently, the therapeutic efficacy of traditional chemotherapeutic drugs for non-small cell lung cancer (NSCLC) was largely limited as the results of the resistance of NSCLC cells to these drugs, including paclitaxel [1], gemcitabine [2] and cisplatin (DDP) [3]. Especially, cisplatin was the most common first-line chemotherapeutic drug for NSCLC [4], and improving DDP chemosensitivity in NSCLC became extremely important and necessary [5, 6]. Cancer stem cells (CSCs) were recently identified as a subgroup of tumor initiating cells in NSCLC [7, 8], which held the ability to self-renew and differentiated into the heterogeneous lineages of cancer cells in response to continuous DDP stimulation [7, 8], and rendered NSCLC cells with resistance to further DDP treatment [9, 10]. Aside from that, DDP pressure also promoted enrichment of CSCs in NSCLC [11]. Based on the above information, a series of strategies to eliminate CSCs in NSCLC microenvironment had been developed to neutralize chemoresistance [10, 12], but the effectiveness was limited because of a lack of knowledge on the detailed mechanisms of DDP-induced CSCs gathering.

Circular RNAs (CircRNAs) were closely associated with NSCLC progression [13] and DDP resistance [14, 15]. CircRNA CDR1as served as an oncogene and promoted NSCLC progression [16, 17]. In addition, CircRNA CDR1as also participated in the regulation of drug resistance in cancer treatment, and inhibition of circRNA CDR1as sensitized breast cancer cells to 5-fluorouracil (5-FU) [18]. Of note, the role of circRNA CDRas in the regulation of DDP chemoresistance was controversial [19–21]. On the one hand, circRNA CDR1as inhibited DDP resistance in ovarian cancer [21] and bladder cancer [20], on the other, circRNA CDR1as increased DDP chemoresistance in lung adenocarcinoma [19], suggesting that circRNA CDR1as regulated DDP resistance in a cancer type dependent manner, but no publications reported the involvement of circRNA CDR1as in DDP chemoresistance in NSCLC. Furthermore, circRNA CDR1as regulated cell stemness, and the existed data reported that circRNA CDR1as maintained the undifferentiated status of mesenchymal stem cells (MSCs) [22].

CircRNAs often regulated cell biological functions by acting as a “RNA sponge” to inhibit MicroRNAs (miRNAs) in a competing endogenous RNA (ceRNA) manner. Specifically, miR-641 was identified as a tumor suppressor in NSCLC [23, 24], and researchers found that miR-641 inhibited NSCLC progression by targeting murine double minute 2 (MDM2) [24] and cyclin-dependent kinase 6 (CDK6) [23], respectively. Notably, previous work indicated that miR-641 could be negatively regulated by circRNA CDR1as in osteoarthritis (OA) chondrocytes [25], indicating that miR-641 might also be the potential downstream target of circRNA CDR1as in NSCLC cells. In addition, miR-641 regulated drug resistance (Erlotinib) in NSCLC [26], but it is still unclear whether miR-641 involved in the regulation of DDP resistance in NSCLC. Furthermore, Homeobox protein Hox-A9 (HOXA9) was closely related with cisplatin resistance in bladder cancer [27], and HOXA9 modulated cancer stem cell properties in pancreatic cancer [28] and glioblastoma [29]. Interestingly, HOXA9 could be negatively regulated by miR-641 in osteosarcoma cells [30], and the online starBase software (http://starbase.sysu.edu.cn/) predicted that miR-641 potentially targeted the 3′ untranslated regions (UTRs) of HOXA9 mRNA.

Collectively, the above information enlightened us that circRNA CDR1as/miR-641/HOXA9 pathway might be crucial for the regulation of cell stemness and DDP chemoresistance in NSCLC, hence this study concentrated on investigating the underlying mechanisms of cisplatin chemoresistance and cell stemness in NSCLC, which will shed light on the discovery potential therapeutic agents to rescue drug sensitivity in chemo-resistant NSCLC cells.

## Materials and methods

### Cell culture and cisplatin treatment

Human NSCLC cell lines (A549, H1299 and Calu6) and HEK-293T cells were purchased from American Type Culture Collections (ATCC, USA), and cisplatin resistant sub-line A549/DDP were obtained from the Resistant Cancer Cell Line (RCCL) collection (http://www.kent.ac.uk/stms/cmp/RCCL/RCCLabout.html). Besides, the acquired cisplatin resistant H1299 (H1299/DDP) and Calu6 (Calu6/DDP) were inducted based on the procedures provided by the previous publication [31]. Briefly, the cells were exposed to continuous low-dose cisplatin stimulation in a step-wise manner, the cisplatin concentrations ranged from 2 μg/ml to 16 μg/ml. The above cells were cultivated in RPMI-1640 medium (Gibco, USA) containing 10% fetal bovine serum under the standard conditions with humidified atmosphere at 37 °C and 5% CO_2_.

### Vectors transfection

The overexpression vectors for circRNA CDR1as (OE-CDR1as) and HOXA9 (OE-HOXA9) were designed and constructed by Vazamy (China), and the short harpin RNA (shRNA) for circRNA CDR1as (KD-CDR1as) and small interfering RNA (siRNA) for HOXA9 (KD-HOXA9) were obtained from Sangon Biotech (Shanghai, China). Besides, miR-641 mimic and inhibitor were also obtained (Sangon Biotech, Shanghai, China). The above vectors were delivered into NSCLC cells by using a commercial Lipofectamine 2000 reagent (Invitrogen, CA, USA) based on the protocols provided by the manufacturer.

### Cell counting kit-8 (CCK-8) assay

Cell proliferation was measured by using a commercial CCK-8 assay kit (YEASEN, Shanghai, China) according to the manufacturer’s protocol. Briefly, the NSCLC cells were administered with different treatments, and incubated with the CCK-8 reaction solution for 2 h. After that, the optical density (OD) values were measured at the wavelength of 450 nm to evaluate cell proliferation abilities.

### Trypan blue staining method

Cell viability was measured by using the commercial trypan blue staining solution purchased from Sigma-Aldrich (USA). The NSCLC cells were stained with the 0.4% trypan blue solution for 20 min, the dead cells were stained with blue. Cell viability was calculated by using the following formula: cell viability (%) = (total cell number − dead blue cell number)/total cell number × 100%.

### Real-Time qPCR

A TRIzol reagent (Invitrogen, USA) was employed to extract the total RNA from NSCLC cells according to manufacturer’s instruction. After that, the expression levels of circRNA CDR1as, miR-641, HOXA9, SOX2, OCT4 and Nanog were determined at transcriptional level by conducting the experimental procedures provided by the previous study [18]. The primer sequences for Real-Time qPCR were listed in Additional file 1: Table S1.

### Western Blot analysis

The commercial RIPA lysis buffer purchased from Beyotime Technology (Shanghai, China) was employed to extract the total protein from NSCLC cells based on the procedures provided by the manufacturer. Further Western Blot analysis was conducted to examine the expression levels of HOXA9 and β-actin, respectively. The detailed experimental procedures were shown in the previous publication [18]. The primary antibodies against HOXA9 (1:1500, Abcam, UK) and β-actin (1:2000, Abcam, UK). The secondary antibody was also purchased from Abcam (1:2500). Besides, the density analysis for protein bands was conducted by using an ECL system (Thermo Fisher, USA). The relative expressions of HOXA9 were normalized to β-actin.

### Dual-luciferase reporter gene system

The online starBase software (http://starbase.sysu.edu.cn/) was used to predict the targeting sites of miR-641 with circRNA CDR1as and 3′ UTR regions of HOXA9 mRNA, respectively. The targeting sequences in circRNA CDR1as and HOXA9 mRNA were mutated, and the wild-type and mutant circRNA CDR1as and HOXA9 were cloned into the luciferase expressing pMIR-REPOTER vectors. The above vectors were co-transfected with miR-641 mimic into HEK-293T cells, and the relative luciferase activity was examined by using the dual-luciferase reporter assay kit (Promega, USA) combined with luminescence plate reader (Molecular Devices Inc., USA) to validate the targeting mechanisms of miR-641 with circRNA CDR1as and HOXA9 mRNA.

### RNA pull-down assay

The RNA pull-down assay was conducted to validate the binding sites of miR-641 with circRNA CDR1as and 3′ UTR regions of HOXA9 mRNA according to the procedures provided by the previous work [32]. In brief, the biotin-labeled circRNA CDR1as and HOXA9 probes were designed and constructed by Sangon Biotech (Shanghai, China). The above probes-streptavidin Dynabeads were next incubated with NSCLC cell lysates at 30 °C overnight. Finally, the crosslinking complexes were disassociated and following Real-Time qPCR was performed to evaluate miR-641 enrichment.

### Colony formation assay

The colony formation abilities of NSCLC cells were determined by using the common colony formation assay according to the procedures from previous study [33]. Briefly, the NCSLC cells were pre-transfected with different vectors and administered with DDP. The cells were seeded onto 6-well plates for 14 days, and the colonies containing at least 10 cells were photographed and counted under light microscope (ThermoFisher Scientific, MA, USA).

### Annexin V-FITC/Propidium Iodide (PI) double stain method for cell apoptosis

Cell apoptosis was examined by using the Annexin V-FITC/PI apoptosis detection kit (BD Bioscience, USA) according to the manufacturer’s protocol. The NSCLC cells were double-stained with Annexin V-FITC and PI for 35 min at room temperature without light exposure, respectively. After that, a flow cytometer (FCM, ThermoFisher Scientific, MA, USA) was used to measure cell apoptosis ratio.

### Spheroid formation assay

The spheroid formation abilities in NSCLC cells were measured by using the spheroid formation assay method, and the experimental procedures were in line with the previous work. In brief, the DDP-sensitive and resistant NSCLC cells were grown in 24-well plates with MannoCult medium (Stem cell technologies, Canada) containing Proliferation Supplements (Stem cell technologies, Canada) for 10 days at standard culturing conditions. Finally, the cell spheres were photographed and counted under light microscope (ThermoFisher Scientific, MA, USA).

### In vivo xenograft mice models

The 4-6 weeks male athymic BALB/c nude mice were obtained from the Research Animal Center of North Sichuan Medical College, and housed under standard conditions. The DDP-resistant NSCLC cells were diluted and implanted into the dorsal flank regions at the concentration of 2 × 10^7^ per animal, and each group consisted of 5 mice. The tumor volumes were measured every 5 days, and the mice were sacrificed at day 30 post-injection. After that, the tumors were obtained and the tumor weights were measured to evaluate tumorigenicity. All the animal experiments were approved by the Ethics Committee for Animal Experimentation of Affiliated Hospital of North Sichuan Medical College.

### Immunohistochemistry (IHC)

The mice tumor tissues were collected and spliced into 5 μm thickness, and the expressions and localization of Ki67 protein were determined by using the IHC assay according to the experimental procedures provided by the previous studies [34, 35]. The expression levels of Ki67 could be employed to represent proliferative abilities of NSCLC cells in vivo.

### Data collection and analysis

All the associated data in this study were collected and analyzed by using the SPSS 18.0 and GraphPad Prism software (Version 8.0). The comparison between two groups were conducted by using the Student’s *t* test, and the one-way Analysis of Variance (ANOVA) method was utilized to compare the differences among multiple groups. Each experiment repeated at least 3 times, and *P < 0.05 was regarded as statistical significance.

## Results

### The expression status of circRNA CDR1as, miR-641 and HOXA9 in NSCLC cells

**Fig. 1:**
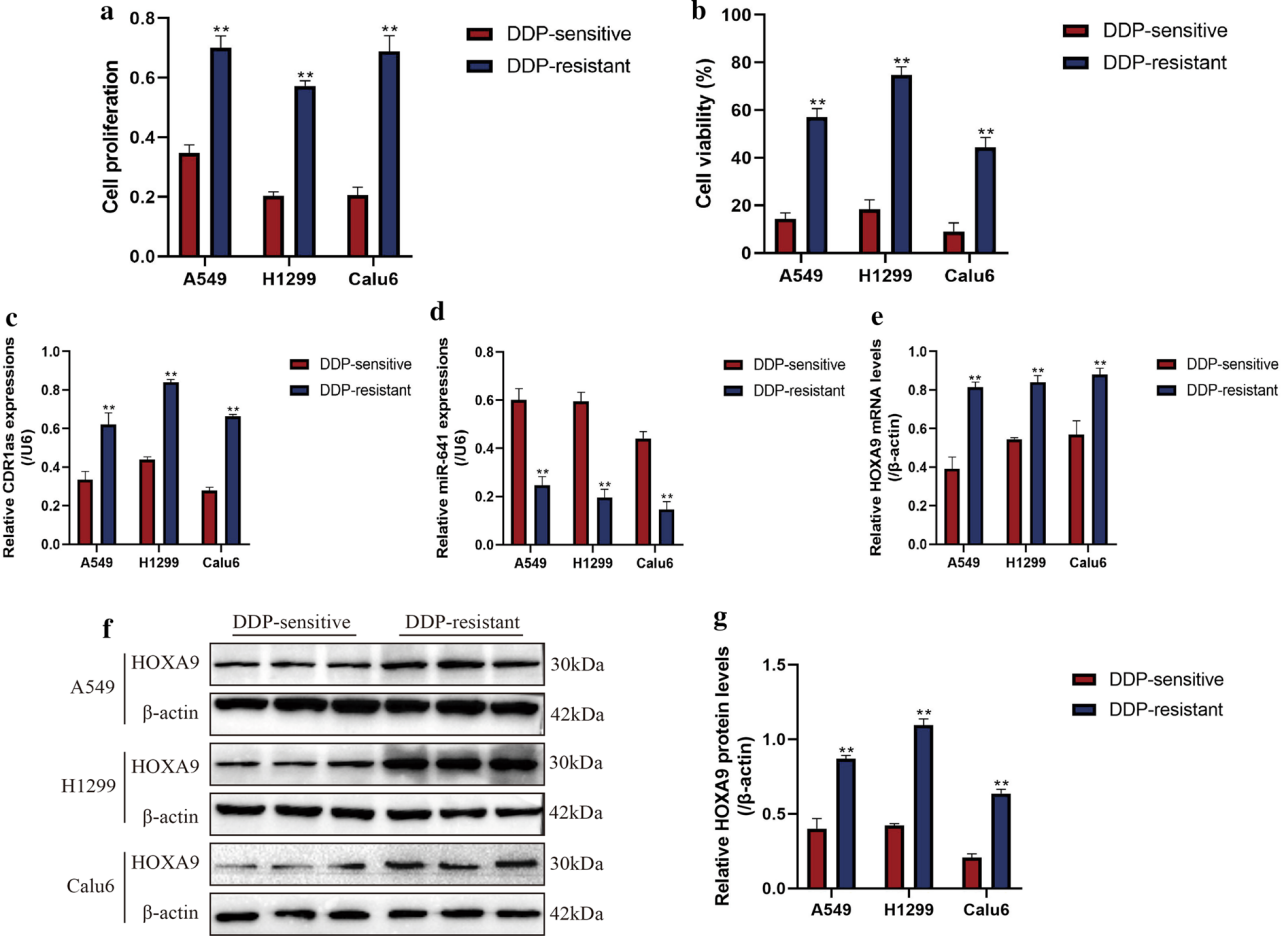
The expression patterns of circRNA CDR1as, miR-641 and HOXA9 in human NSCLC cell lines (A549, H1299 and Calu6) and their paired descendent cisplatin-resistant sub-lines (A549/DDP, H1299/DDP and Calu6/DDP). The above cells were cultured in standard conditions and subsequently stimulated with high-dose cisplatin for 48 h, cell proliferation was measured by **a** CCK-8 assay and **b** trypan blue staining method was employed to detect cell viability. The results indicated that A549/DDP, H1299/DDP and Calu6/DDP were more resistant to high-dose cisplatin treatment compared to the parental DDP-sensitive NSCLC cells. The expression levels of **c** circRNA CDR1as and **e** HOXA9 mRNA were upregulated, while **d** miR-641 was downregulated in DDP-resistant NSCLC cells compared to DDP-sensitive cells, determined by using Real-Time qPCR. **f**, **g** Western Blot results showed that HOXA9 protein levels were increased in DDP-resistant NSCLC cells. Each experiment repeated at least 3 times. **P < 0.01

Aberrant gene expressions were closely related with drug resistance in cancer treatment [36]. Mechanistically, long-term stimulation by cisplatin altered expression patterns of cancer associated genes, which rendered the subgroups of cancer cells with resistance to this drug [36]. The existed literatures highlighted the relevance of circRNA CDR1as, miR-641 and HOXA9 with cisplatin resistance in NSCLC, hence, we investigated whether the expression patterns of circRNA CDR1as, miR-641 and HOXA9 were changed by continuous cisplatin stimulation. To achieve this, human NSCLC cell lines (A549, H1299 and Calu6) and their paired descendent cisplatin-resistant sub-lines (A549/DDP, H1299/DDP and Calu6/DDP) were obtained and cultured under standard conditions. Subsequently, the above cells were subjected to high-dose cisplatin stimulation for 48 h. The CCK-8 (Fig. 1a) and trypan blue assay (Fig. 1b) results indicated that A549/DDP, H1299/DDP and Calu6/DDP were much more resistant to high-dose cisplatin stimulation compared to their parental DDP-sensitive cells, suggesting that the DDP-resistant NSCLC cells were successfully obtained. By analyzing the expression levels of circRNA CDR1as, miR-641 and HOXA9 in the above cells (Fig. 1c–g), we surprisingly found that circRNA CDR1as (Fig. 1c) and HOXA9 mRNA (Fig. 1e) were upregulated, but miR-641 (Fig. 1d) was downregulated in DDP-resistant NSCLC cells compared to the DDP-sensitive NSCLC cells. Consistently, further Western Blot results validated that HOXA9 was high expressed in DDP-resistant NSCLC cells at protein levels (Fig. 1f, g), suggesting that the expression patterns of circRNA CDR1as, miR-641 and HOXA9 were changed in DDP-resistant NSCLC cells, and miR-641 negatively correlated with circRNA CDR1as and HOXA9.

### CircRNA CDR1as positively regulated HOXA9 by sponging miR-641 in DDP-sensitive NSCLC cells

**Fig. 2:**
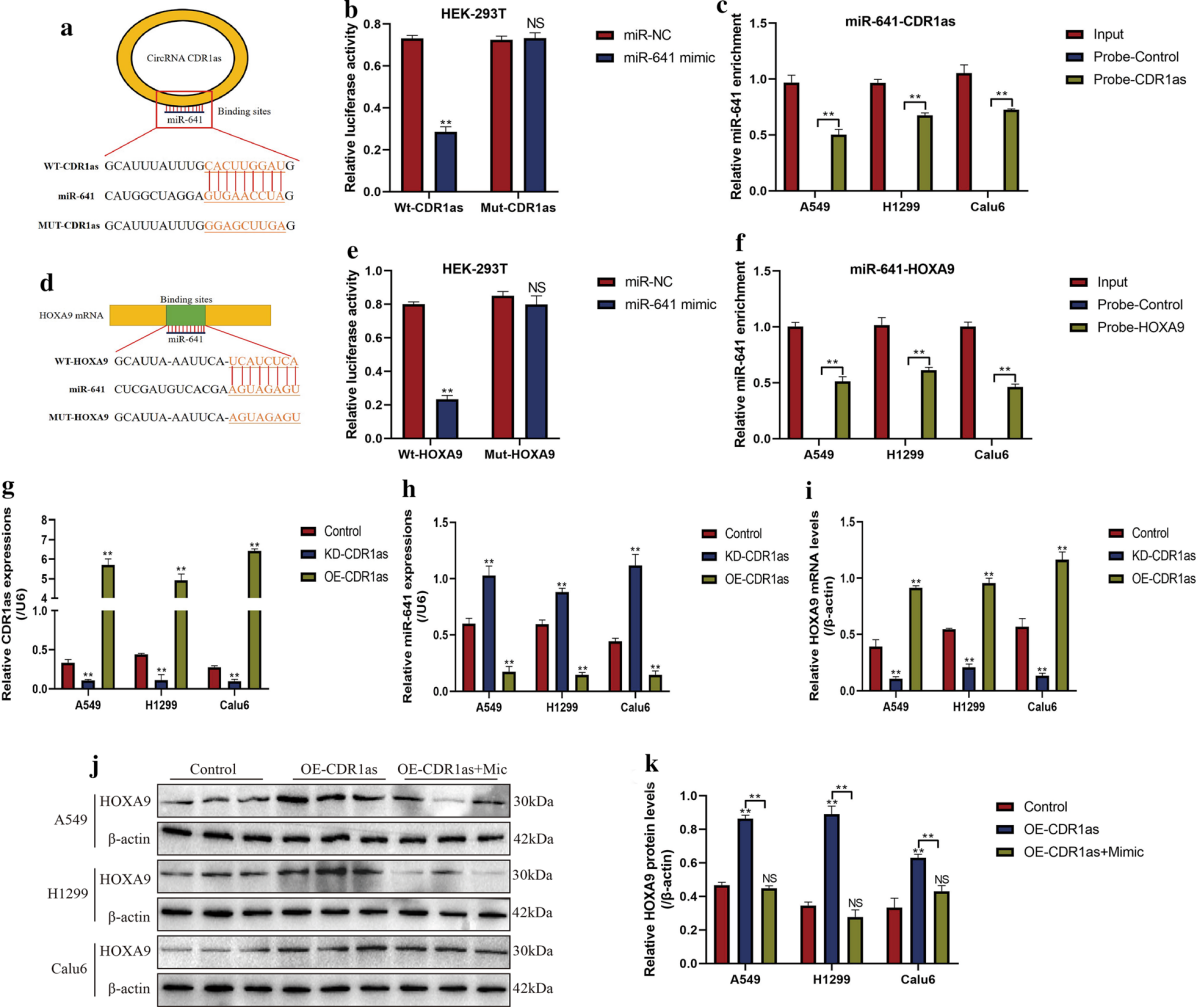
CircRNA CDR1as positively regulated HOXA9 in DDP-sensitive NSCLC cells by sponging miR-641. The online starBase software (http://starbase.sysu.edu.cn/) predicted the binding sites of miR-641 with **a** circRNA CDR1as and **d** 3′ UTR regions of HOXA9 mRNA. Dual-luciferase reporter gene system validated the targeting sites of miR-641 with **b** circRNA CDR1as and **e** 3′ UTR regions of HOXA9 mRNA. RNA pull-down assay results indicated that miR-641 could be enriched by **c** circRNA CDR1as and **f** HOXA9 mRNA probes, respectively. **g** CircRNA CDR1as was successfully silenced and overexpressed in DDP-sensitive NSCLC cells, determined by Real-Time qPCR. CircRNA CDR1as negatively regulated the levels of **h** miR-641 and positively regulated **i** HOXA9 mRNA in NSCLC cells. **j**, **k** Western Blot analysis revealed that circRNA CDR1as inhibited HOXA9 protein levels in NSCLC cells by targeting miR-641. Each experiment repeated at least 3 times. **P < 0.01. “NS” represented no statistical significance

**Fig. 3:**
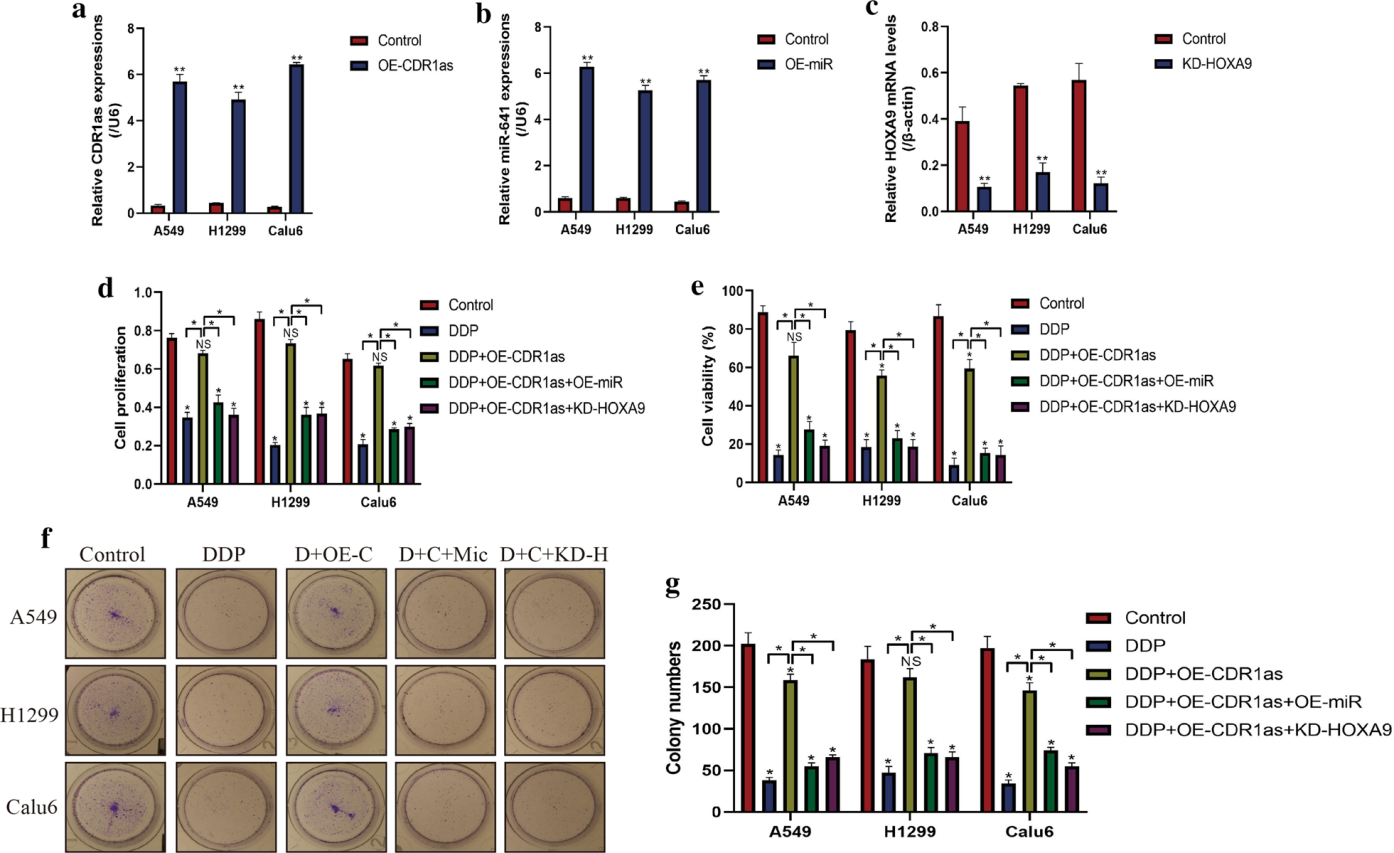
Overexpression of circRNA CDR1as increased DDP chemoresistance in DDP-sensitive NSCLC cells. The **a** circRNA CDR1as overexpression vectors, **b** miR-641 mimic and **c** vectors for HOXA9 ablation were pre-transfected into NSCLC cells, examined by Real-Time qPCR. Subsequently, the NSCLC cells (A549, H1299 and Calu6) were subjected to high-dose DDP for 48 h. **d** CCK-8 assay and **e** trypan blue assay results indicated that upregulated circRNA CDR1as increased DDP chemoresistance in DDP-sensitive NSCLC cells by regulating miR-641/HOXA9 axis. **f**, **g** Colony formation assay results showed that circRNA CDR1as rescued colony formation abilities in DDP-treated NSCLC cells by regulating miR-641/HOXA9 axis. (Note: “D” represented “DDP”, “C” and “OE-C” represented “circRNA CDR1as overexpression”, “Mic” represented “miR-641 mimic” and “KD-H” meant “Knock-down of HOXA9″). *P < 0.05, **P < 0.01. “NS” represented no statistical significance

Previous data suggested that there existed potential regulating mechanisms among circRNA CDR1as, miR-641 and HOXA9 [25, 30], which were validated in this study. Specifically, the online starBase software (http://starbase.sysu.edu.cn/) predicted that miR-641 could bind to circRNA CDR1as (Fig. 2a) and 3′ UTR regions of HOXA9 mRNA (Fig. 2d). Besides, previous data supported that circRNA CDR1as acted as RNA sponge of miR-641 [25], and HOXA9 was the downstream target of miR-641 [30], which rendered the possibility that miR-641 might serve as a “bridge” to combine circRNA CDR1as and HOXA9. The above hypothesis was validated by the following experiments, and we found that CircRNA CDR1as promoted HOXA9 expressions in DDP-sensitive NSCLC cells by targeting miR-641 (Fig. 2). Specifically, the dual-luciferase reporter gene system results showed that miR-641 could bind to both circRNA CDR1as (Fig. 2b) and 3′UTR regions of HOXA9 mRNA (Fig. 2e) in HEK-293T cells, and the RNA pull-down assay validated that miR-641 could be enriched by both circRNA CDR1as (Fig. 2c) and HOXA9 (Fig. 2f) probes in NSCLC cells. In addition, the vectors for circRNA CDR1as overexpression and downregulation were successfully transfected into DDP-sensitive NSCLC cells (Fig. 2g). The results showed that circRNA CDR1as negatively regulated miR-641 (Fig. 2h), while positively regulated HOXA9 mRNA (Fig. 2i) levels in CS-NSCLC cells. Of note, the promoting effects of circRNA CDR1as overexpression on HOXA9 were abrogated by upregulating miR-641 (Fig. 2j, k), suggesting that circRNA CDR1as promoted HOXA9 expressions by competitively binding to miR-641.

### Upregulation of circRNA CDR1as increased cisplatin chemoresistance in DDP-sensitive NSCLC cells by targeting miR-641/HOXA9 axis

Since circRNA CDR1as [18], miR-641 [26] and HOXA9 [27] involved in the regulation of drug resistance, we next investigated whether circRNA CDR1as/miR-641/HOXA9 pathway regulated cisplatin resistance in DDP-sensitive NSCLC cells. To accomplish this, the circRNA CDR1as overexpression vectors (Fig. 3a), miR-641 mimic (Fig. 3b) and short hairpin RNA (shRNA) for HOXA9 (Fig. 3c) were pre-transfected into DDP-sensitive NSCLC cells, respectively. Subsequently, the above cells were stimulated with high-dose cisplatin. The CCK-8 (Fig. 3d) and trypan blue assay (Fig. 3e) results showed that circRNA CDR1as overexpression rescued cell proliferation and viability in DDP-sensitive NSCLC cells treated with cisplatin, which were reversed by both overexpressing miR-641 and silencing HOXA9. In addition, upregulation of circRNA CDR1as also increased colony formation abilities in cisplatin treated NSCLC cells, which were abrogated by miR-641 overexpression and HOXA9 silence (Fig. 3f, g). The above results indicated that circRNA CDR1as overexpression increased resistance of DDP-sensitive NSCLC cells to cisplatin by regulating miR-641/HOXA9 axis.

### CircRNA CDR1as ablation sensitized DDP-resistant NSCLC cells to cisplatin by regulating miR-641/HOXA9 axis

**Fig. 4:**
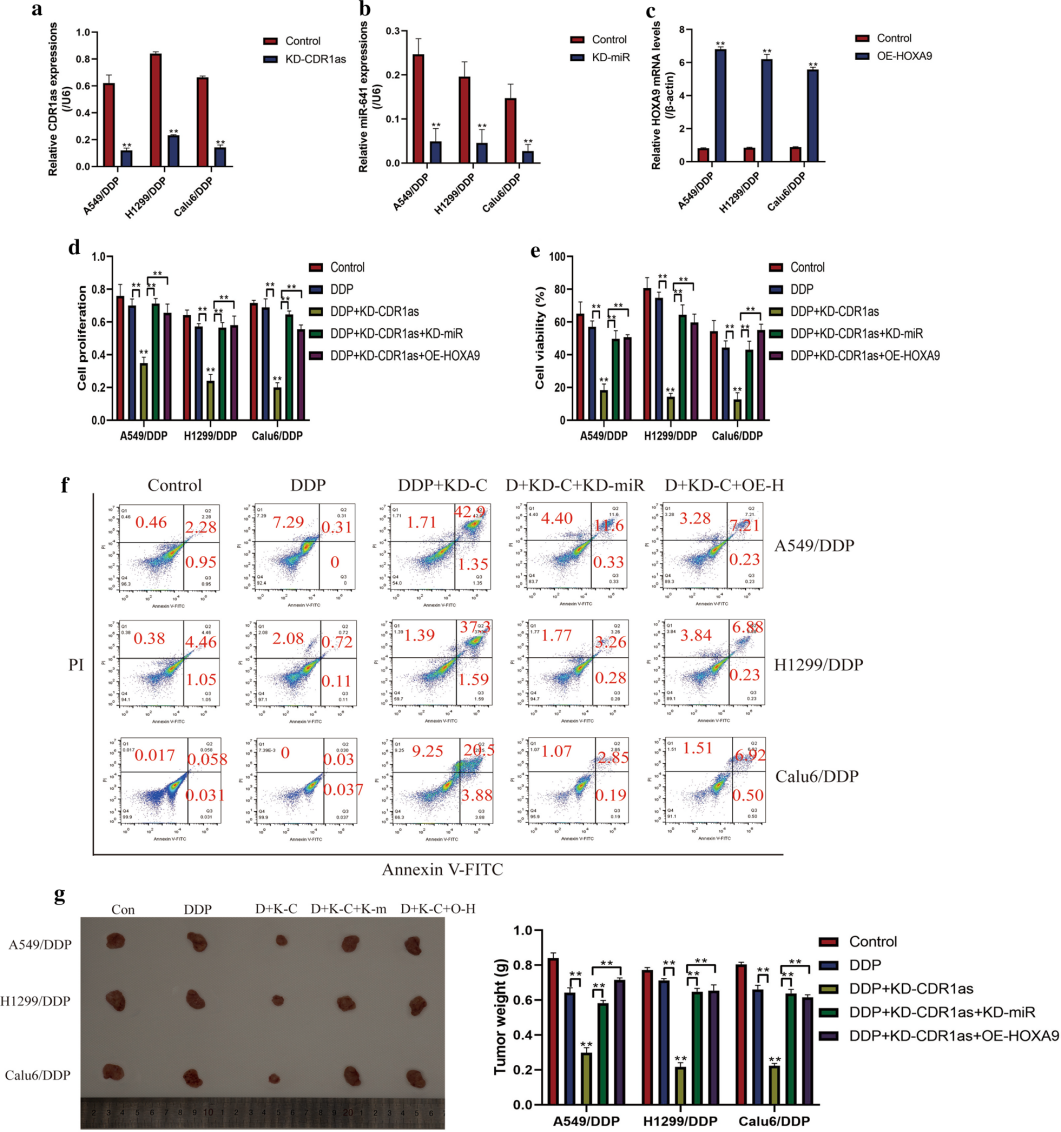
Knock-down of circRNA CDR1as sensitized DDP-resistant NSCLC cells to by regulating miR-641/HOXA9 axis. The **a** circRNA CDR1as downregulation vectors, **b** miR-641 inhibitor and **c** HOXA9 overexpression vectors were successfully delivered into DDP-resistant NSCLC cells, examined by Real-Time qPCR. **d** CCK-8 assay and **e** trypan blue assay results indicated that circRNA CDR1as deficiency aggravated the inhibiting effects of DDP on DDP-resistant NSCLC cell proliferation and viability by downregulating HOXA9 through upregulating miR-641. **f** Annexin V-FITC/PI double stain method combined with flow cytometer (FCM) revealed that knock-down of circRNA CDR1as increased apoptosis ratio in DDP treated A549/DDP, H1299/DDP and Calu6/DDP cells by regulating miR-641/HOXA9 axis. **g** The xenograft tumor bearing mice models were established to evaluate the tumorigenicity of NSCLC cells in vivo. (Note: “D” represented “DDP”, “KD-C” represented “circRNA CDR1as silence”, “KD-miR” represented “miR-641 ablation” and “OE-H” meant “overexpression of HOXA9”). Each experiment repeated at least 3 times. *P < 0.05, **P < 0.01. “NS” represented no statistical significance

The vectors for CircRNA CDR1as downregulation (Fig. 4a), miR-641 inhibitor (Fig. 4b) and HOXA9 overexpression (Fig. 4c) were next pre-transfected into DDP-resistant NSCLC cells (A549/DDP, H1299/DDP and Calu6/DDP), and the cells were subsequently exposed to high-dose DDP stimulation. As expected, the CCK-8 assay (Fig. 4d) and trypan blue assay (Fig. 4e) results showed that knock-down of CircRNA CDR1as enhanced the inhibiting effects of DDP on cell proliferation and viability in DDP-resistant NSCLC cells, which were reversed by downregulating miR-641 and overexpressing HOXA9. In addition, the apoptosis ratio of DDP-resistant NSCLC cells was measured by Annexin V-FITC/PI double stain method (Fig. 4f). The results showed that deficiency of CircRNA CDR1as significantly increased apoptosis ratio in cisplatin treated DDP-resistant NSCLC cells, and the promoting effects of circRNA CDR1as on DDP-resistant NSCLC cell death were abrogated by silencing miR-641 and upregulating HOXA9 (Fig. 4f). Furthermore, by establishing the tumor-bearing mice models, we proved that knock-down of circRNA CDR1as aggravated the inhibiting effects of DDP on tumorigenesis by targeting miR-641/HOXA9 axis in vivo (Fig. 4g). Consistently, silencing circRNA CDR1as combined DDP stimulation decreased the expression levels of Ki67 in mice tumor tissues (Additional file 2: Figure S1). The above data suggested that silence of circRNA CDR1as sensitized DDP-resistant NSCLC cells to DDP by regulating miR-641/HOXA9 axis.

### CircRNA CDR1as regulated stemness properties in NSCLC cells by targeting miR-641/HOXA9 axis

**Fig. 5:**
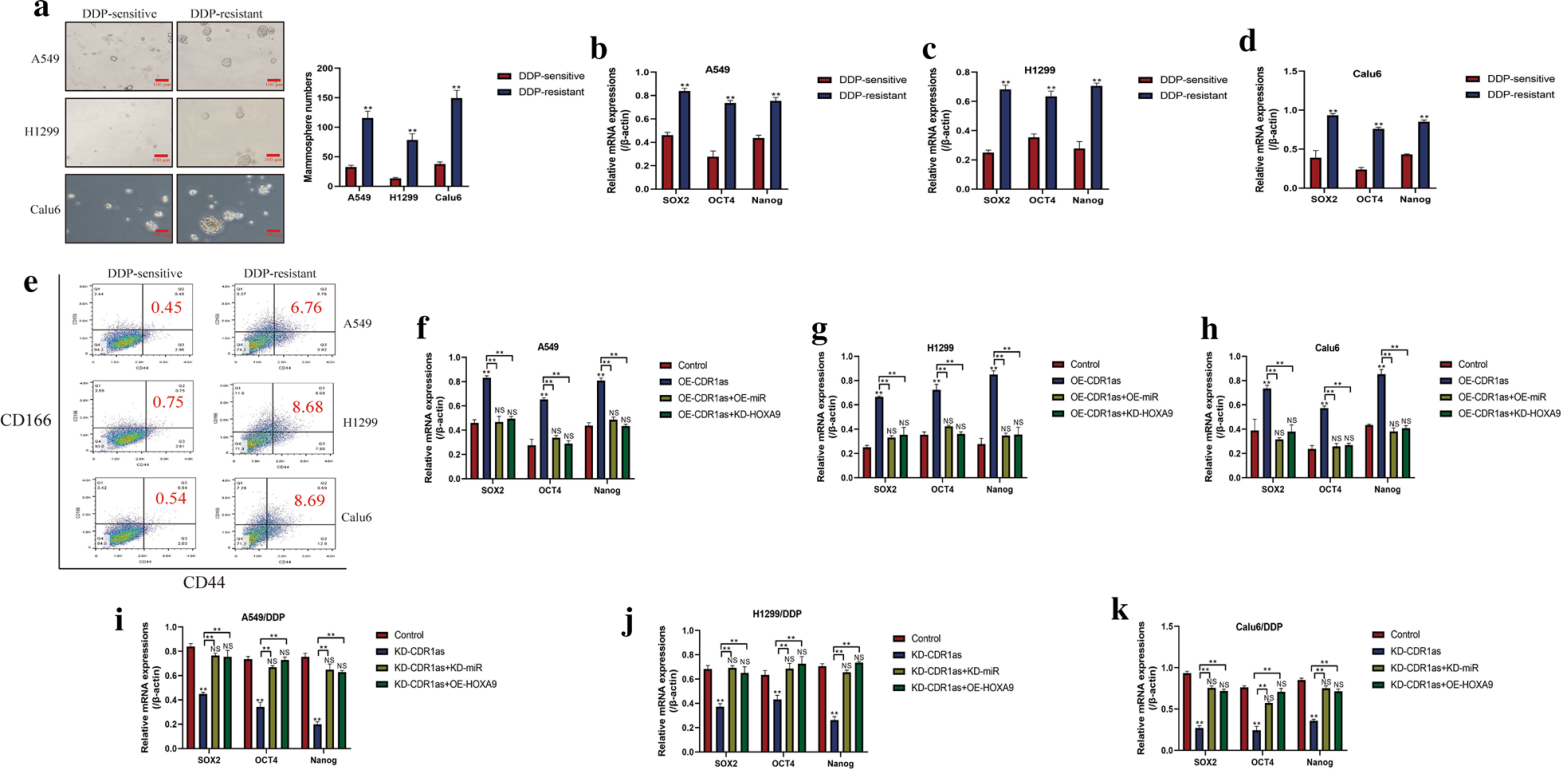
CircRNA CDR1as/miR-641/HOXA9 axis regulated stemness of NSCLC cells in vitro. **a** The spheroid formation assay results indicated that DDP-resistant NSCLC cells tended to form spheres in contrast with their corresponding parental DDP-sensitive NSCLC cells. **b**–**d** The mRNA levels of SOX2, OCT4 and Nanog were upregulated in DDP-resistant NSCLC cells compared to DDP-sensitive NSCLC cells, examined by Real-Time qPCR. **e** Flow cytometer was used to measure the proportion of CD44^+^CD166^+^ cells in NSCLC cells. **f**–**h** Upregulation of circRNA CDR1as promoted stemness associated signatures expression in DDP-sensitive NSCLC cells by regulating miR-641/HOXA9 axis. **i**–**k** Knock-down of circRNA CDR1as inhibited stemness properties in DDP-resistant NSCLC cells by targeting miR-641/HOXA9 axis. Each experiment repeated at least 3 times. *P < 0.05, **P < 0.01. “NS” represented no statistical significance

Enrichment of CSCs in NSCLC microenvironment contributed to drug resistance and cancer recurrence, and the existed information hinted that circRNA CDR1as/miR-641/HOXA9 pathway regulated cell stemness. Based on this, we found that circRNA CDR1as promoted stemness in NSCLC cells by targeting miR-641/HOXA9 axis (Fig. 5). Specifically, by culturing the DDP-resistant and sensitive NSCLC cells under standard conditions, the spheroid formation assay results showed that DDP-resistant NSCLC cells were prone to form spheres compared to the corresponding DDP-sensitive NSCLC cells (Fig. 5a). Consistently, the Real-Time qPCR results indicated that the mRNA levels of SOX2, OCT4 and Nanog were much higher in DDP-resistant NSCLC cells, instead of DDP-sensitive NSCLC cells (Fig. 5b–d), and CD44^+^CD166^+^ cells tended to be enriched in DDP-resistant NSCLC cells, compared to their parental DDP-sensitive counterparts (Fig. 5e). In addition, we found that overexpression of circRNA CDR1as increased the mRNA levels of SOX2, OCT4 and Nanog in DDP-sensitive NSCLC cells, which were reversed by upregulating miR-641 and downregulating HOXA9 (Fig. 5f–h). Similarly, further experiments validated that knock-down of circRNA CDR1as inhibited stemness properties in DDP-resistant NSCLC cells, and the inhibiting effects of circRNA CDR1as ablation on stemness in DDP-resistant NSCLC cells were abrogated by knocking down miR-641 and overexpressing HOXA9 (Fig. 5i–k), implying that circRNA CDR1as regulated stemness in NSCLC cells by targeting miR-641/HOXA9 axis in vitro.

## Discussion

Cisplatin (DDP) was the first-line chemotherapeutic drug for NSCLC treatment in clinic [3], however, the therapeutic efficacy of DDP was seriously limited as the results of DDP chemoresistance generated by NSCLC cells [5, 6]. Therefore, researchers currently focused on investigating the underlying mechanisms of DDP chemoresistance to develop potential therapeutic agents to rescue DDP chemosensitivity in NSCLC cells [5, 6]. Recent publications reported that circular RNA (circRNA)-microRNA (miRNA)-mRNA networks were closely associated with cancer progression [37, 38] and drug resistance [14] in NSCLC, and this study identified a novel circRNA CDR1as/miR-641/HOXA9 pathway played an important role in the regulation of DDP chemoresistance in NSCLC. Mechanistically, circRNA CDR1as and HOXA9 were high-expressed, while miR-641 was low-expressed in DDP-resistant NSCLC cells compared to their parental DDP-sensitive NSCLC cells, which were in line with the previous work [19, 26, 27]. Additionally, previous data indicated that there existed potential regulatory mechanisms among circRNA CDR1as, miR-641 and HOXA9 [25, 30], and we validated that circRNA CDR1as positively regulated HOXA9 expressions in NSCLC cells by sponging miR-641.

The role of circRNA CDR1as in the regulation of DDP chemoresistance was controversial according to the cancer types [19–21], and we found that circRNA CDR1as increased DDP chemoresistance in NSCLC cells, which were in line with the previous work [19]. In addition, miR-641 [26] and HOXA9 [27] also participated in the regulation of drug resistance in cancers, and miR-641/HOXA9 axis could be regulated by circRNA CDR1as in NSCLC cells, which rendered the possibility that circRNA CDR1as regulated DDP chemoresistance in NSCLC cells by targeting miR-641/HOXA9 axis. Based on the above information, we next investigated circRNA CDR1as/miR-641/HOXA9 pathway regulated DDP chemoresistance in DDP-sensitive and resistant NSCLC cells, respectively. As expected, the results showed that upregulation of circRNA CDR1as increased cell proliferation, viability and colony formation abilities in DDP-sensitive NSCLC cells treated with high-dose DDP, which were abrogated by overexpressing miR-641 and downregulating HOXA9, implying that upregulated circRNA CDR1as increased DDP chemoresistance in DDP-sensitive NSCLC cells by targeting miR-641/HOXA9 axis. Consistently, knock-down of circRNA CDR1as inhibited cell proliferation and viability, but increased cell apoptosis ratio in DDP-resistant NSCLC cells treated with high-dose DDP, which were all reversed by downregulating miR-641 and upregulating HOXA9, indicating that circRNA CDR1as ablation increased DDP chemosensitivity in NSCLC cells by downregulating HOXA9 through upregulating miR-641.

Cancer stem cells (CSCs) held the ability to self-renew and differentiate into the heterogeneous lineages of cancer cells [7, 8], which contributed to drug resistance and NSCLC recurrence [9, 10]. Mechanistically, long-term chemotherapeutic drug stimulation caused genes mutation in NSCLC cells, which led to the generation and enrichment of a subgroup of NSCLC cells with stemness properties to antagonize DDP treatment [11]. Expectedly, this study found that DDP-resistant NSCLC cells were prone to form spheres, enrich CD44^+^CD166^+^ cells and accompanied by higher expression levels of stemness signatures compared to DDP-sensitive NSCLC cells, indicating that CSCs were enriched in DDP-resistant NSCLC cells and in accordance with the previous work [11]. Additionally, circRNA CDR1as involved in the regulation of cell stemness [22], and we verified that overexpressed circRNA CDR1as promoted CSCs enrichment in DDP-sensitive NSCLC cells, and circRNA CDR1as inhibited stemness properties in DDP-resistant NSCLCs, indicating that circRNA CDR1as positively regulated cell stemness in NSCLC cells. Interestingly, HOXA9 also regulated CSCs [28, 29], and our data suggested that circRNA CDR1as regulated CSCs enrichment in NSCLC cells by regulating miR-641/HOXA9 axis. The above results hinted that targeting circRNA CDR1as/miR-641/HOXA9 pathway was novel to eliminate CSCs in DDP-resistant NSCLC cells. However, this study merely investigated the role of circRNA CDR1as/miR-641/HOXA9 pathway in regulating NSCLC cell stemness in vitro, and further xenograft animal models are still needed to validate the above cellular results in vivo.

## Conclusion

The present study validated that knock-down of circRNA CDR1as sensitized DDP-resistant NSCLC cells to DDP and inhibited cell stemness properties by downregulating HOXA9 through upregulating miR-641. This study will shed light on the discovery of potential therapeutic agents to increase DDP chemosensitivity in NSCLC cells.

## Supplementary information

**Additional file 1: Table S1.** Primer sequences for Real-Time qPCR.

**Additional file 2: Additional file 1. Figure S1.** The expressions and localization of Ki67 protein in mice tumor tissues were examined by using the immunohistochemistry (IHC) assay, and the results indicated that knock-down of circRNA CDR1as combined with DDP stimulation decreased Ki67 protein levels in mice tumor tissues. Each experiment repeated at least 3 times.

## Data Availability

The datasets supporting the conclusions of this article are included within the article.
