# Supplementary material for: CircRNA CDR1as/miR-641/HOXA9 pathway regulated stemness contributes to cisplatin resistance in non-small cell lung cancer (NSCLC)
Source: Cancer Cell Int. 2020 Jul 6;20:289. doi: 10.1186/s12935-020-01390-w (PMC7339514; doi:10.1186/s12935-020-01390-w)
Supplement: Supplementary file 1 — Additional file 1: Table S1. Primer sequences for Real-Time qPCR. [file 12935_2020_1390_MOESM1_ESM.docx]

**Table S1. Primer sequences for Real-Time qPCR.**

| Gene | Primer sequences (strand) |
| --- | --- |
| β-actin | Forward: 5’-CTCCATCCTGGCCTCGCTGT-3’  Reverse: 5’-GCTGCTACCTTCACCGTTCC-3’ |
| U6 | Forward: 5’-GACTATCATATGCTTACCGT-3’  Reverse: 5’-GGGCAGGAAGAGGGCCTAT-3’ |
| CDR1as | Forward: 5’-TAGTACGTCGTGCCCTGA-3’  Reverse: 5’-CACTTGACGTGCAGCATC-3’ |
| miR-641 | Forward: 5’-TTATACTCTCACCATTTGGATC-3’  Reverse: 5’-TGACAAGATTTTACATCAAGAA-3’ |
| HOXA9 | Forward: 5’-CTTACCCAAGCTTCACTCACC-3’  Reverse: 5’- AAGAGGCCTGGTGCTACTAC-3’ |
| OCT4 | Forward: 5’-AGCGATCAAGCAGCGACTA-3’  Reverse: 5’-GGAAAGGGACCGAGGAGTA-3’ |
| SOX2 | Forward: 5’-CATCACCCACAGCAAATGAC-3’  Reverse: 5’-CAAAGCTCCTACCGTACCACT-3’ |
| Nanog | Forward: 5’-GCAGGCAACTCACTTTATCC -3’  Reverse: 5’-CCCACAAATCACAGGCATAG-3’ |
