# Supplementary figures and images for: CircRNA CDR1as/miR-641/HOXA9 pathway regulated stemness contributes to cisplatin resistance in non-small cell lung cancer (NSCLC)
Source: Cancer Cell Int. 2020 Jul 6;20:289. doi: 10.1186/s12935-020-01390-w (PMC7339514; doi:10.1186/s12935-020-01390-w)

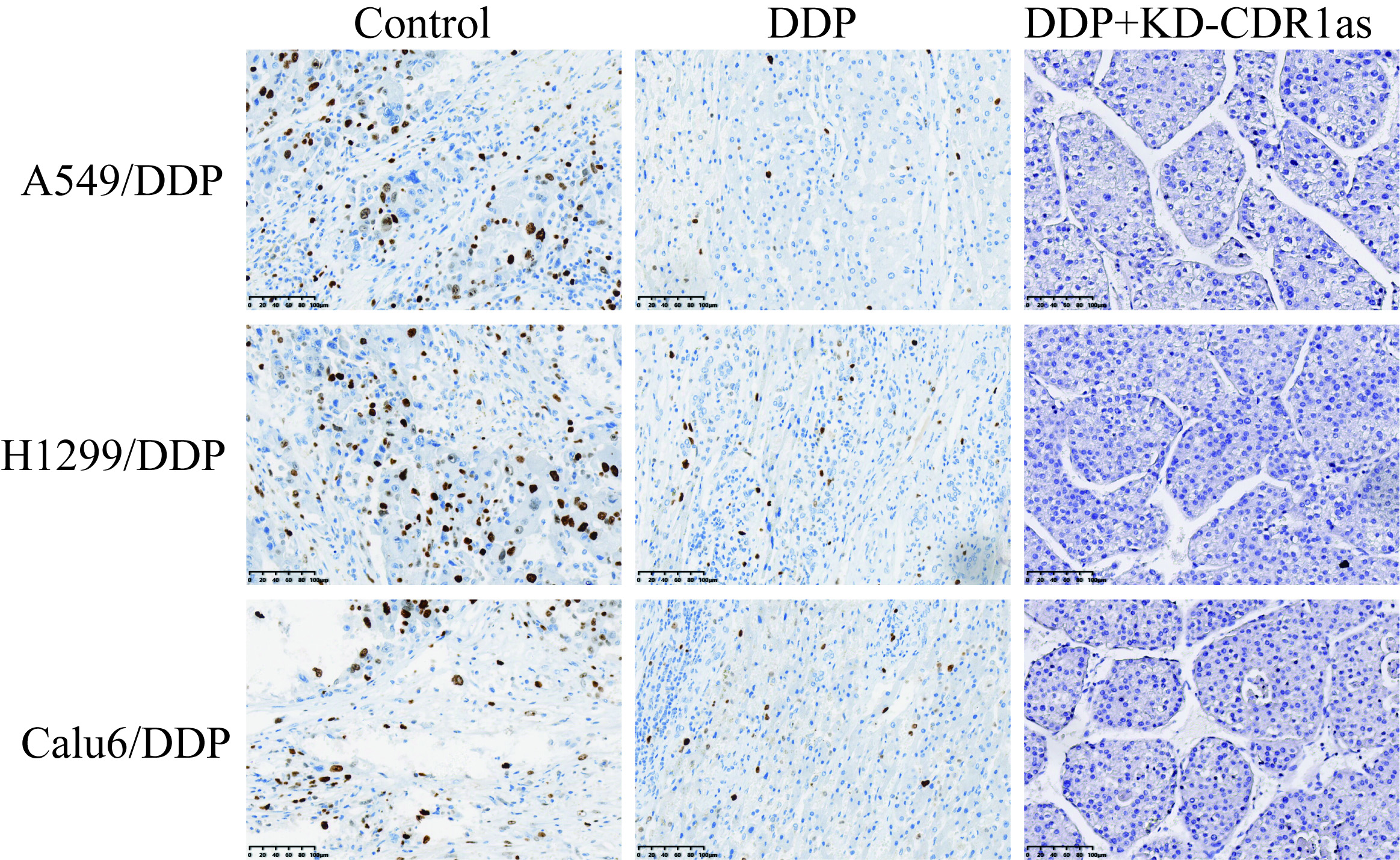

Supplement: Supplementary file 2 — Additional file 2: Additional file 1. Figure S1. The expressions and localization of Ki67 protein in mice tumor tissues were examined by using the immunohistochemistry (IHC) assay, and the results indicated that knock-down of circRNA CDR1as combined with DDP stimulation decreased Ki67 protein levels in mice tumor tissues. Each experiment repeated at least 3 times. [file 12935_2020_1390_MOESM2_ESM.jpg]
